# Supplementary material for: Exploration of the metabolomic mechanisms of postmenopausal hypertension induced by low estrogen state
Source: eLife. 2025 Jan 16;13:RP101701. doi: 10.7554/eLife.101701 (PMC11737871; doi:10.7554/eLife.101701)
Supplement: Supplementary file 30. [file elife-101701-supp30.docx]

***The legends for the main figures***

**Figure 1** Research flowchart.

**Figure 2** Establishment of animal Models. (A) Serum estrogen concentration, n = 8 per group (B). SBP of animal, n = 8 per group (C) DBP, n = 8 per group. (D) PP of animal, n = 8 per group. ***p < 0.001.

**Figure 3** Overview of aorta metabolomics related to estrogen deprivation. (A) Metabolite classification statistics chart. (B) Venn diagram of differential metabolites. (C-I) Bar chart of the expression levels of the most promising differential metabolites. (J) SAM analysis of differential metabolites.

**Figure 4** Correlation analysis of samples and differential metabolites. (A) Pearson correlation analysis heatmap of samples. (B) Hierarchical clustering tree diagram of samples. (C) Hierarchical clustering heatmap of samples and metabolites.

**Figure 5** Overview of dimension reduction analysis. (A) PCA scatter plot. (B) PLS-DA scatter plot. (C) sPLS-DA scatter plot. (D) VIP score of sPLS-DA model. (E) VIP score of RF model (F) OOB error for the random forest tree model.

**Figure 6** Overview of subgroup dimensionality reduction analysis 1. (A) Volcano map of differential metabolites between the Sham group and OVX group. (B) Volcano map of differential metabolites between the OVX group and OV +E group.

**Figure 7** Overview of subgroup dimensionality reduction analysis 2. (A) Hierarchical clustering dendrogram between the Sham group and OVX group. (B) Hierarchical clustering dendrogram between the OVX group and OVX+E group. (C) Hierarchical clustering heatmap between the Sham group and OVX group. (B) Hierarchical clustering heatmap between the OVX group and OVX+E.

**Figure 8** The subgroup dimensionality reduction analysis. (A) PCA scatter plot between the Sham group and OVX group. (B) PCA scatter plot between the OVX group and OVX+E group. (C) PLS-DA scatter plot between the Sham group and OVX group. (D) PLS-DA scatter plot between the OVX group and OVX+E group. (E) OPLS-DA scatter plot between the Sham group and OVX group. (F) OPLS-DA scatter plot between the OVX group and OVX+E group. (G) OPLS-DA model permutations test between the Sham group and OVX group. (H) OPLS-DA models permutations test between the OVX group and OVX+E group. (I) VIP score of OPLS-DA model between the Sham group and OVX group. (J) VIP score of OPLS-DA model between the OVX group and OVX+E group.

**Figure 9** Screening differential metabolites using RF, EBAM, and SAM methods. (A) VIP patterns based on RF model between the Sham group and OVX group. (B) VIP patterns based on RF model between the OVX group and OVX + E group. (C, D) Volcano plots for subgroup comparison based on EBAM method. (E, F) Screening of differential metabolites between subgroups based on SAM method.

**Figure 10** Expression patterns of metabolites. (A) Metabolite expression patterns associated with ovariectomy. (B) Metabolite expression patterns related to estrogen supplementation. (C) Metabolite expression patterns associated with increased estrogen concentration. (D) Metabolite expression patterns associated with L-AABA.

**Figure 11** Screening of typical biomarkers. (A) ROC curve of L-AABA between the Sham group and OVX group. (B) ROC curve of L-AABA between the OVX group and OVX+E group. (C) Multivariate ROC curve based exploratory analysis between the Sham and OVX group. (D) Multivariate ROC curve based exploratory analysis between the OVX group and OVX+E group. (E) Average importance ranking of multivariate models for biomarkers between the Sham and OVX group. (F) Average importance ranking of multivariate models for biomarkers between the OVX and OVX + E group.

**Figure 12** Comprehensive Analysis of Differential Metabolites. (A) Classification pie chart of differential metabolites. (B) Metabolic enrichment analysis of differential metabolites. (C) Enzyme enrichment analysis of differential metabolites. (D) Enrichment analysis of rat specific metabolite pathways.

***The legends for the supplementary figures***

**Figure 3 - Figure supplement 1** Normalization processing rendering. (A) Distribution map of samples before and after normalization treatment. (B) Distribution map of metabolites before and after normalization treatment.

**Figure 3 - Figure supplement 2** Multi-class Significance Analysis model.

**Figure 3 - Figure supplement 3** A heatmap of metabolite correlations.

**Figure 5 - Figure supplement 1** Dimension reduction analysis of the three groups. (A) PCA plot among the three groups (B) PCA scree plot among the three groups. (C) PLS-DA plot among the three groups. (D) VIP score of OPLS-DA model among the three groups. (E) 5-fold CV of OPLS-DA model among the three groups. (F) OPLS-DA models permutations test among the three groups. (G) sPLS-DA plot among the three groups. (H) sPLS-DA classification error rates plot among the three groups.

**Figure 6 - Figure supplement 1** Subgroup normalization processing rendering. (A) Distribution map of metabolites before and after normalization treatment between the Sham and OVX group. (B) Distribution map of samples before and after normalization treatment between the Sham and OVX group. (C) Distribution map of metabolites before and after normalization treatment between the OVX group and OVX+E group. (D) Distribution map of samples before and after normalization treatment between the OVX group and OVX+E group.

**Figure 8-figure supplement 1** The subgroup dimensionality reduction analysis. (A) PCA plot between the Sham group and OVX group. (B) PCA plot between the OVX group and OVX+E group. (C) PLS-DA plot between the Sham group and OVX group. (D) PLS-DA plot between the OVX group and OVX+E group. (E) PLS-DA model permutations test between the Sham group and OVX group. (F) PLS-DA models permutations test between the OVX group and OVX+E group. (G) OPLS-DA model permutations test between the Sham group and OVX group. (H) OPLS-DA models permutations test between the OVX group and OVX+E group.

**Figure 8-figure supplement 2** The subgroup random forest model (A) The random forest classification and OOB error plot between the Sham group and OVX group. (B) The random forest classification and OOB error plot between the OVX group and OVX+E group.

**Figure 11-figure supplement 1**The error classification of the multivariate ROC curve. (A) The misclassifications between the OVX and Sham groups. (B) The misclassifications between the OVX and OVX+E groups.
